# Reviewing Mobile Apps for Teaching Human Anatomy: Search and Quality Evaluation Study

**DOI:** 10.2196/64550

**Published:** 2025-02-14

**Authors:** Guadalupe Esmeralda Rivera García, Miriam Janet Cervantes López, Juan Carlos Ramírez Vázquez, Arturo Llanes Castillo, Jaime Cruz Casados

**Affiliations:** 1 Tecnológico Nacional de México, Instituto Tecnológico Superior de Pánuco Pánuco, Veracruz Mexico; 2 Facultad de Medicina de Tampico “Dr. Alberto Romo Caballero” de la Universidad Autónoma de Tamaulipas Tampico, Tamaulipas Mexico

**Keywords:** anatomy, Google Play, mobile health, mHealth, Mobile App Rating Scale, MARS

## Abstract

**Background:**

Mobile apps designed for teaching human anatomy offer a flexible, interactive, and personalized learning platform, enriching the educational experience for both students and health care professionals.

**Objective:**

This study aimed to conduct a systematic review of the human anatomy mobile apps available on Google Play, evaluate their quality, highlight the highest scoring apps, and determine the relationship between objective quality ratings and subjective star ratings.

**Methods:**

The Mobile App Rating Scale (MARS) was used to evaluate the apps. The intraclass correlation coefficient was calculated using a consistency-type 2-factor random model to measure the reliability of the evaluations made by the experts. In addition, Pearson correlations were used to analyze the relationship between MARS quality scores and subjective evaluations of MARS quality item 23.

**Results:**

The mobile apps with the highest overall quality scores according to the MARS (ie, sections A, B, C, and D) were Organos internos 3D (anatomía) (version 4.34), Sistema óseo en 3D (Anatomía) (version 4.32), and VOKA Anatomy Pro (version 4.29). To measure the reliability of the MARS quality evaluations (sections A, B, C, and D), the intraclass correlation coefficient was used, and the result was “excellent.” Finally, Pearson correlation results revealed a significant relationship (*r*=0.989; *P*<.001) between the quality assessments conducted by health care professionals and the subjective evaluations of item 23.

**Conclusions:**

The average evaluation results of the selected apps indicated a “good” level of quality, and those with the highest ratings could be recommended. However, the lack of scientific backing for these technological tools is evident. It is crucial that research centers and higher education institutions commit to the active development of new mobile health apps, ensuring their accessibility and validation for the general public.

## Introduction

### Background

At present, there is a wealth of research on mobile apps focused on various aspects and areas of health, such as musculoskeletal injuries [1]; chronic disease management [2]; pediatric disease care [3]; medication management [4]; oral hygiene [5]; asthma [6]; pediatric ear, nose, and throat surgery [7]; low back pain [8]; neurodegenerative disorders [9]; coronary arteries [10]; neurorehabilitation [11]; nutrition, anemia, and preeclampsia [12]; cancer [13,14]; cerebrovascular diseases [15]; childhood obesity [16]; diabetes [17]; tuberculosis [18]; fibromyalgia [19]; dementia [20]; chronic kidney disease [21]; and epilepsy [22], among others. These mobile health management apps have transformed the way people access and manage information about their well-being, enabling everything from vital signs monitoring to chronic disease management. This advancement in mobile apps not only benefits patients but also opens new possibilities in health education. In particular, mobile apps for teaching human anatomy have become valuable resources that complement the learning and understanding of the structures and functions of the human body. Similar to personal health apps, these tools are designed with interactive features such as 3D models and detailed simulations that enhance the educational experience for medical students and health care professionals.

The justification for this study is based on three important approaches: (1) the technological approach regarding the use of mobile apps for teaching human anatomy, (2) the pedagogical approach, and (3) the quality evaluation approach of the apps. These approaches together provide a solid foundation to justify the importance of the study.

### Technological Approach

The first approach involved reviewing and analyzing scientific publications on the use of mobile apps in teaching human anatomy, with the aim of understanding their results. This approach highlights how mobile technology has revolutionized access to knowledge, enabling students to learn in an accessible and practical manner. In teaching human anatomy, mobile apps with 3D models and simulations facilitate immersive and effective learning, complementing and even enhancing traditional methods in health sciences. One of the studies presented the software Road to Birth, developed by the University of Newcastle, which was designed to teach midwifery students at a Midwestern US university about the dynamic concepts of maternal anatomy and physiology during an obstetrics module. The students used Road to Birth, and 66% of them reported an increase in their knowledge, valuing the software as a useful and practical learning resource [23]. Another study used the mobile app AR in Anatomy, developed by the authors; which allows users to dynamically explore various parts of the human body in 3D, enhancing the educational experience [24]. Similarly, apps such as Anat_Hub, developed by faculty and researchers from the Departments of Computer Science and Medical Sciences at the University of the Western Cape; a mobile app with augmented reality (AR) to improve learning about the musculoskeletal system’s anatomy, received positive evaluations. User results indicated that the anatomy system could effectively enhance student engagement and retention of anatomical concepts [25]. In addition, another study conducted with undergraduate health sciences students at the University of Cape Town analyzed the impact of an AR mobile app on learning motivation. The study included 78 students, evaluating motivation levels before and after using the app. The results showed that its use increased motivation, improving aspects such as attention, satisfaction, and confidence [26]. In the field of neuroanatomy, the use of mobile AR facilitated the understanding of complex concepts, increasing academic performance and reducing cognitive load among students [27]. Similarly, HuMAR, developed by researchers affiliated with Murdoch University and Universiti Utara Malaysia; an AR-based prototype for learning skeletal structure, demonstrated high satisfaction among students, highlighting the system’s usability and functionality [28]. However, although cadavers remain the gold standard in anatomy teaching, there are financial, ethical, and supervisory limitations. Another study compared the effectiveness of virtual reality, AR, and tablet-based devices in teaching cranial anatomy. A total of 59 students participated who were randomly assigned to one of the 3 learning methods. The results suggest that these technologies can effectively complement anatomical teaching [29]. On the other hand, a recent study presented a human anatomy learning system based on AR using a marker on a mobile platform to capture images and merge them with data from an SQLite database. This system allows for interactive visualization of the human body or its organs in 3D. An evaluation conducted with high school and medical students demonstrated that the app facilitates anatomy learning more effectively due to its ability to provide interactive 3D representations through AR [30]. Another example is AEducaAR, an app developed by researchers affiliated with the University of Bologna, which combines AR with a 3D-printed anatomical model to improve anatomy teaching for medical students. Its effectiveness was evaluated with a group of 62 second-year students, comparing its use to traditional learning methods with anatomical atlas books. Although there were no significant differences in objective test results between the two methods, students expressed enthusiasm for AEducaAR in a survey, valuing its potential to motivate learning and enhance the 3D understanding of anatomical structures. This tool could also prepare students to use advanced medical technologies in their future careers [31]. In addition, a relevant study presents 10 mobile apps for teaching human anatomy, where the results indicate that the technological designs studied exhibit a high degree of usability [32]. Another study analyzed 325 anatomy mobile apps and outlined their features to facilitate dissemination in the academic field. It showcases a broad, diverse, and affordable market for human anatomy mobile apps that can complement students’ education [33].

### Pedagogical Approach

Medical students frequently face challenges in understanding anatomy through the images found in textbooks [34], which are flat and lack interactivity. In contrast, mobile apps for learning human anatomy can serve as a complementary resource for learning this discipline, offering students the opportunity to interact with content more deeply than in a conventional dissection room. Although traditional cadaver-based teaching remains the preferred learning method [35], anatomy education continues to face numerous challenges, including limited practical hours for students and instructors, restricted access, and the high cost of cadavers and artificial models [36]. The use of mobile apps for learning human anatomy offers significant advantages, such as immediate and continuous access to information anytime and anywhere. These apps include interactive 3D designs that allow for a detailed exploration of the human body. Furthermore, they are often updated regularly and are generally more affordable than traditional textbooks, making them accessible to a broader audience.

### Quality Evaluation Approach

The third approach focuses on evaluating the quality of human anatomy mobile apps. The importance of evaluating these apps lies in the fact that higher-rated apps can serve as academic support for medical students, health care professionals, and general users. A specific methodology is required to evaluate the quality of health mobile apps. In this context, the Mobile App Rating Scale (MARS) methodology has been used. Various studies have used the MARS to assess health apps targeting a variety of conditions, such as chronic kidney disease and end-stage renal disease [37], chronic lung diseases [38], stress management [39], psoriasis [40], gastrointestinal diseases [41], pain management [42], oral hygiene [43,44], nutrition [45], genetics and genomics [46], food allergies or intolerances [47], deafness and hearing impairment [48], low back pain [49,50], neurological conditions [51], peritoneal dialysis [52], diabetes [53], COVID-19 [54], cancer [55], anticoagulation [56], dementia [57], specialized diets [58], toric intraocular lenses [59], epilepsy [60], depression [61], coronary diseases [62], dyslexia [63], autism spectrum disorder [64], nutrition [65], and pediatric palliative care [66].

This study aimed to (1) identify human anatomy mobile apps available on the Google Play store, which uses the Android operating system, covering 70.87% of the global mobile operating system market [67]; (2) evaluate these apps using the MARS, which considers engagement, functionality, aesthetics, information, subjective quality, and app specificity; (3) present the human anatomy mobile apps with the highest ratings on the MARS; and (4) determine the correlation between the objective MARS quality rating and the subjective MARS rating by health care professionals (ie, item 23).

## Methods

### Overview

This study was cross-sectional, as it collected data at a single time point without follow-up over time, evaluated the correlation between variables, and provided descriptive data [68-70]. The study was conducted following the guidelines of the STROBE (Strengthening the Reporting of Observational Studies in Epidemiology) initiative, which aims to improve the communication of results from observational studies among authors, editors, and readers of scientific publications, focusing primarily on cohort, case-control, and cross-sectional studies [71].

### Selection Criteria for Human Anatomy Mobile Apps

From April 1 to May 30, 2024, an extensive search for mobile apps was conducted. The search term used was *anatomy*. The inclusion criteria for mobile apps were as follows: (1) available on the Google Play store; (2) related to human anatomy; (3) available in English or Spanish; (4) user rating ≥4.3 to ensure a minimum level of acceptance and satisfaction among users; (5) free to use—an essential factor in educational contexts where students or universities may face budget constraints; and (6) download count exceeding 100,000.

The exclusion criteria were as follows: (1) duplicate apps, either because of different versions or alternative names but containing the same content; and (2) apps not updated for 2 or more years.

The PRISMA (Preferred Reporting Items for Systematic Reviews and Meta-Analyses) methodology was used to select human anatomy–teaching apps. This methodology is used in systematic reviews and meta-analyses to ensure transparency and rigor in the selection and analysis of relevant studies. The phases were identification, selection, eligibility, and inclusion.

During the identification phase, an exhaustive search for mobile apps dedicated to teaching human anatomy was conducted. In the filtering phase, duplicate apps were discarded. In the eligibility phase, the characteristics of these apps were analyzed to discard those that did not meet the previously established inclusion criteria, and an additional exclusion criterion was applied. Finally, in the inclusion phase, the apps that met all eligibility requirements were integrated for analysis and evaluation.

All selected apps were recorded in Excel (version 2016; Microsoft Corporation) with the following characteristics: app name, identification screen, languages, star rating, total downloads, developer, Android version, last update date, and features.

### Evaluation of Mobile Apps

#### Overview

We used the MARS, developed by Stoyanov et al [72], which has been widely used to evaluate the design and usability of mobile health apps. The MARS consists of 3 dimensions. The first dimension is an objective tool based on 4 main components: engagement (section A), functionality (section B), aesthetics (section C), and information quality (section D). The second dimension assesses subjective quality (section E), and the third dimension evaluates the perceived effectiveness (section F).

Each section of the MARS has several items. An item refers to a specific element, question, or unit of evaluation within a questionnaire or survey. Section A comprises 5 items and evaluates whether the app is engaging, interesting, customizable, interactive, and targeted at a specific population. Section B comprises 4 items and focuses on the app’s performance, ease of use, navigation, and gesture design. Section C comprises 3 items and examines the app’s design, graphics, and visual appeal. Section D comprises 7 items and analyzes the accuracy of information description, objectives, quality and quantity of information, visual information quality, credibility, and scientific evidence base of the evaluated app. The 4 objective sections of MARS (ie, A, B, C, and D) encompass 19 items. The average scores of sections A, B, C, and D represent the overall MARS quality score [72].

Section E comprises 4 items and focuses on the evaluator’s personal perception of the app and typically includes items that ask about the likelihood of recommending the app, the probability of the user continuing to use the app, and the overall perception of its quality. Finally, section F comprises 6 items and focuses on how health care professionals perceive the impact of the app on their knowledge, attitudes, intentions, and behaviors related to health.

#### Evaluation Instrument Scale

To evaluate each item, a 5-point Likert scale was used, ranging from 1 to 5 (1=inadequate, 2=poor, 3=acceptable, 4=good, and 5=excellent). A MARS score of more than 3 points indicates acceptable quality.

#### Selection of Evaluators

A total of 10 evaluators were selected, all health care professionals from the Faculty of Medicine of Tampico Dr Alberto Romo Caballero at the Universidad Autónoma de Tamaulipas, Tampico, Tamaulipas, Mexico. The inclusion criteria were (1) being a top-performing student in the final year of medical school and (2) having a mobile phone with the Android operating system to download human anatomy teaching apps from Google Play.

#### Evaluation Process

Before starting the evaluation of anatomy-related apps, it was necessary to train the evaluators in the use of the MARS. For this, the authors of this study convened the 10 selected evaluators at the library of the Faculty of Medicine of Tampico Dr Alberto Romo Caballero at the Universidad Autónoma de Tamaulipas, Tampico, Tamaulipas, Mexico, to present a training video in English by Stoyanov et al [72]. Following the video presentation, a training exercise was suggested for all evaluators using an app called Anatomymaster. This app, also focused on human anatomy, was not included in the study sample, as it did not meet the requirement of having a user rating ≥4.3.

The evaluators downloaded and tested the trial app for at least 10 minutes before completing the MARS web-based questionnaire. If any individual evaluation score varied by at least 2 points, the evaluators discussed it to reach a consensus, ensuring a uniform understanding of each item.

After completing the trial exercise, the 10 selected evaluators evaluated the 18 human anatomy mobile apps during June 2024. Each evaluator was provided with a list of apps, which they downloaded and used for 10 minutes before completing a web-based evaluation instrument designed based on the MARS. Each item from the different sections was rated using a Likert scale (1-5). The collected data were initially recorded in Excel.

### Statistical Analysis

#### Overview

Statistical analyses, including the calculation of the intraclass correlation coefficient (ICC) and Pearson correlation coefficient, were performed using SPSS (version 29.0.2.0; IBM Corp).

#### Intraclass Correlation Coefficient

The ICC was used using a random effects model of 2 factors with consistency to measure the overall agreement between the quantitative measurements obtained by different evaluators [73]. The ICC ranges from 0 to 1, with 0 indicating a total lack of reliability among evaluators and 1 representing perfect reliability. According to the 95% CI for ICC estimation, values below 0.5 are considered “poor” reliability, those between 0.5 and 0.75 are considered “moderate,” those between 0.75 and 0.9 are considered “good,” and those above 0.90 are rated as “excellent” [74].

The individual ICC was calculated for each of the sections A, B, C, and D. The arithmetic means of each section were used to calculate the ICC for the overall MARS quality score (ie, sections A, B, C, and D). In section A, 900 data points were considered, accounting for 10 evaluators, 5 items, and 18 mobile apps. In section B, 720 data points were used (ie, 10 evaluators, 4 items, and 18 mobile apps). In section C, 540 data points were analyzed (ie, 10 evaluators, 3 items, and 18 mobile apps). Finally, in section D, 1080 data points were considered (ie, 10 evaluators, 6 items, and 18 mobile apps). In this last section, item 19 was excluded because of missing values; therefore, only 6 items were considered instead of 7. For each MARS section (ie, A, B, C, and D), the arithmetic mean and SD were calculated.

#### Pearson Correlation

The statistical technique of Pearson correlation was used to evaluate the relationship between the MARS quality scores (ie, sections A, B, C, and D) and the subjective item 23 from section E. The software used was SPSS.

### Ethical Considerations

This study did not involve experiments on humans, animals, or the collection of sensitive personal data. It focused exclusively on evaluating publicly available mobile applications on Google Play through a structured analysis conducted by health care professionals. No direct interaction with developers or users of the applications took place, and no private or identifiable information was accessed or stored.

This type of research, which does not involve mental health e-communities or sensitive data, does not require institutional review board approval. Furthermore, the activities were carried out following the institutional policies and local guidelines of the Universidad Autónoma de Tamaulipas, Mexico, for observational research and technological product reviews.

This decision aligns with international ethical standards and aims to ensure transparency and accountability in the evaluation of publicly available technological products.

## Results

### Selection Criteria for Anatomy Mobile Apps

**Figure 1:**
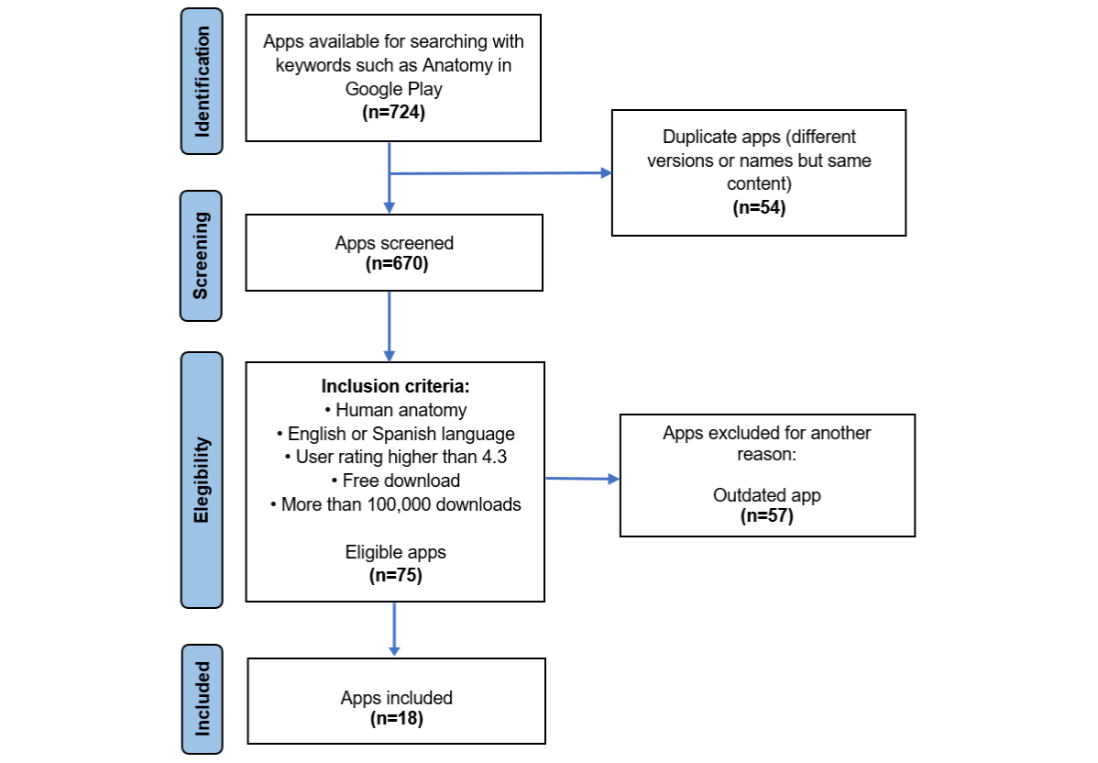
Flowchart of the selection process.

Figure 1 shows the PRISMA diagram applied to the selection of mobile apps; a total of 724 apps were identified in the Google Play store under the search criterion *anatomy*. In total, 54 duplicate apps were eliminated either because they had the same name or different names but the same content, leaving 670 in the screening phase. In the eligibility phase, the inclusion criteria were applied, where only 75 apps met these characteristics. In the same way, 57 apps that were more than 2 years old without receiving updates from the developer were excluded, finally leaving 18 apps in the inclusion phase.

In Table 1, the names of the selected apps, their identification screens, developer names, required Android operating system version, and the date of the last update are presented. All the listed mobile apps focus on human anatomy, are available in English or Spanish, have a user rating above 4.3, can be downloaded for free, and have more than 100,000 downloads.

**Table 1:** Characteristics of selected mobile apps (Google Play, 2024).

App name	Identification screen	Developer	Android version	Last update
Anatomy Learning-Anatomía 3D	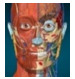	3D Medical OU	7.0 and later versions	June 16, 2024
Complete Anatomy 2024	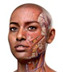	3D4Medical from Elsevier	7.0 and later versions	May 28, 2024
Biodigital Human-3D Anatomy	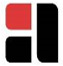	BioDigital	5.0 and later versions	February 28, 2024
Anatomía–Atlas 3D	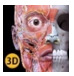	Catfish Animation Studio	8.0 and later versions	August 21, 2023
Anatomyka-Anatomy 3D	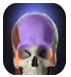	Woodoo Art s.r.o.	5.1 and later versions	November 17, 2023
VOKA 3d Anatomy and Physiology	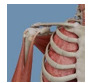	Factory of innovations and solutions LLC	8.0 and later versions	January 27, 2024
Organos internos 3D (anatomía)	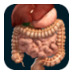	Ing Víctor Michel González Galván	5.1 and later versions	November 1, 2023
Teach Me Anatomy	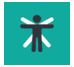	TeachMeSeries Ltd	7.0 and later versions	May 22, 2024
3D Bones and Organs (Anatomy)	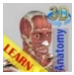	Education Mobile	5.1 and later versions	September 3, 2023
Esqueleto|Anatomía 3D	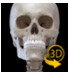	Catfish Animation Studio	8.0 and later versions	August 21, 2023
Visual Anatomy Lite	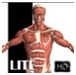	Education Mobile	4.4 and later versions	August 9, 2023
Gray’s Anatomy-Anatomy Atlas	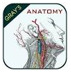	SEStudio	4.4 and later versions	March 1, 2023
El cuerpo humano en 3D	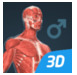	Mozaik Education	5.0 and later versions	May 30, 2024
e-Anatomy	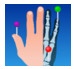	IMAIOS SAS	5.0 and later versions	June 11, 2024
Sistema muscular 3D (Anatomía)	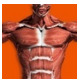	Ing. Víctor Michel González Galván	5.1 and later versions	November 27, 2023
Sistema óseo en 3D (Anatomía)	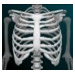	Ing. Víctor Michel González Galván	5.1 and later versions	November 6, 2023
Anatomy by Muscle & Motion	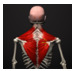	Muscle and Motion	5.0 and later versions	April 2, 2024
Flashcards de Daily Anatomy	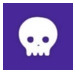	Kenhub	5.0 and later versions	September 21, 2023

### Evaluation of Mobile Apps

#### MARS Overall Quality Scores

The average overall MARS quality score (ie, sections A, B, C, and D) was rated as “good” (mean 4.02, SD 0.20) [75]. The 3 mobile apps with the highest overall MARS quality scores (ie, averages of sections A, B, C, and D) were Organos internos 3D (anatomía) (mean 4.34, SD 0.29), Sistema óseo en 3D (Anatomía) (mean 4.32, SD 0.28), and VOKA Anatomy Pro (mean 4.29, SD 0.28). In contrast, the apps with the lowest overall MARS quality scores were Anatomy–3D Atlas (mean 3.66, SD 0.27), Complete Anatomy 2024 (mean 3.73, SD 0.32), and Visual Anatomy Lite (mean 3.80, SD 0.28). The average overall MARS quality scores are listed in Table 2.

**Table 2:** Average Mobile App Rating Scale quality scores (sections A, B, C, and D).

App name	Section A—engagement (mean 3.69, SD 0.20), mean (SD)	Section B—functionality (mean 4.36, SD 0.22), mean (SD)	Section C— aesthetics (mean 4.14, SD 0.21), mean (SD)	Section D—information (mean 3.90, SD 0.23), mean (SD)	Arithmetic average A, B, C, and D (mean 4.02, SD 0.20), mean (SD)
Organos internos 3D (anatomía)	3.96 (0.19)	4.65 (0.19)	4.43 (0.23)	4.30 (0.13)	4.34 (0.29)
Sistema óseo en 3D (Anatomía)	3.98 (0.28)	4.63 (0.19)	4.47 (0.15)	4.22 (0.17)	4.32 (0.28)
VOKA 3 d Anatomy and Physiology	3.94 (0.11)	4.60 (0.24)	4.37 (0.12)	4.23 (0.05)	4.29 (0.28)
Anatomy Learning-Anatomía 3D	3.88 (0.15)	4.55 (0.13)	4.27 (0.31)	4.20 (0.24)	4.22 (0.28)
Flashcards de Daily Anatomy	3.82 (0.28)	4.55 (0.10)	4.30 (0.20)	4.05 (0.10)	4.18 (0.32)
Teach Me Anatomy	3.76 (0.24)	4.48 (0.15)	4.40 (0.20)	3.95 (0.15)	4.15 (0.35)
Anatomyka- Anatomía 3D	3.74 (0.23)	4.50 (0.14)	4.23 (0.42)	4.00 (0.11)	4.12 (0.32)
3D Bones and organs (Anatomy)	3.72 (0.33)	4.53 (0.05)	4.03 (0.12)	3.90 (0.18)	4.04 (0.35)
El cuerpo humano en 3D	3.64 (0.21)	4.38 (0.21)	4.20 (0.26)	3.92 (0.10)	4.03 (0.32)
Sistema muscular 3D (Anatomía)	3.84 (0.27)	4.33 (0.15)	4.13 (0.42)	3.83 (0.05)	4.03 (0.24)
Biodigital Human-3D Anatomy	3.66 (0.31)	4.30 (0.08)	4.10 (0.44)	3.80 (0.06)	3.97 (0.29)
Anatomy by Muscle & Motion	3.66 (0.24)	4.45 (0.13)	4.07 (0.38)	3.60 (0.13)	3.94 (0.40)
e-Anatomy	3.54 (0.36)	4.20 (0.28)	4.07 (0.15)	3.87 (0.08)	3.92 (0.29)
Gray’s Anatomy-Anatomy Atlas	3.58 (0.32)	4.18 (0.29)	3.93 (0.06)	3.72 (0.08)	3.85 (0.26)
Esqueleto|Anatomía 3D	3.48 (0.13)	4.10 (0.34)	4.00 (0.10)	3.75 (0.15)	3.83 (0.28)
Visual Anatomy Lite	3.44 (0.05)	4.08 (0.35)	3.97 (0.06)	3.70 (0.06)	3.80 (0.28)
Complete Anatomy 2024	3.34 (0.09)	4.05 (0.31)	3.93 (0.06)	3.58 (0.08)	3.73 (0.32)
Anatomía–Atlas 3D	3.36 (0.05)	4.00 (0.08)	3.70 (0.30)	3.57 (0.23)	3.66 (0.27)

#### MARS Section Scores (Sections A, B, C, and D)

The mean (SD) for the “engagement” section (ie, section A) was 3.69 (0.20). The 3 top-rated apps in this section were Sistema óseo en 3D (Anatomía) (mean 3.98, SD 0.28), Organos internos 3D (anatomía) (mean 3.96, SD 0.19), and VOKA Anatomy Pro (mean 3.94, SD 0.19). In contrast, the 3 apps with the lowest scores in section A were Complete Anatomy 2024 (mean 3.34, SD 0.09), Anatomy–3D Atlas (mean 3.36, SD 0.05), and Visual Anatomy Lite (mean 3.44, SD 0.05).

For the “functionality” section (ie, section B), the mean (SD) was 4.36 (0.22). The top-rated apps were Organos internos 3D (anatomía) (mean 4.65, SD 0.19), Sistema óseo en 3D (Anatomía) (mean 4.63, SD 0.19), and VOKA Anatomy Pro (mean 4.60, SD 0.24). Conversely, the 3 apps with the lowest scores in this section were Anatomy–3D Atlas (mean 4.00, SD 0.08), Complete Anatomy 2024 (mean 4.05, SD 0.31), and Visual Anatomy Lite (mean 4.08, SD 0.35).

Regarding the “aesthetics” section (ie, section C), the mean (SD) was 4.14 (0.21). The highest-rated apps were Sistema óseo en 3D (Anatomía) (mean 4.47, SD 0.15), Organos internos 3D (anatomía) (mean 4.43, SD 0.23), and Teach Me Anatomy (mean 4.40, SD 0.20). In contrast, the lowest-scoring apps in this section were Anatomy–3D Atlas (mean 3.70, SD 0.30), Complete Anatomy 2024 (mean 3.93, SD 0.06), and Gray’s Anatomy-Anatomy Atlas (mean 3.93, SD 0.06).

For the “information quality” section (ie, section D), the mean (SD) was 3.90 (0.23). The highest-rated apps were Organos internos 3D (anatomía) (mean 4.30, SD 0.13), VOKA Anatomy Pro (mean 4.23, SD 0.05), and Sistema óseo en 3D (Anatomía) (mean 4.22, SD 0.17). In contrast, the lowest-scoring apps in this section were Anatomy–3D Atlas (mean 3.57, SD 0.23), Complete Anatomy 2024 (mean 3.58, SD 0.08), and Anatomy by Muscle and Motion (mean 3.60, SD 0.13; Table 2).

#### Subjective Quality Evaluation (Section E) and Perceived Effectiveness (Section F)

The general mean (SD) for the “subjective quality” section (ie, section E) was 3.63 (0.22). The 3 top-rated mobile apps in this section were VOKA Anatomy Pro (mean 3.95, SD 0.10), Organos internos 3D (anatomía) (mean 3.93, SD 0.17), and Sistema óseo en 3D (Anatomía) (mean 3.88, SD 0.22). Conversely, the 3 apps with the lowest scores in this section were Anatomy–Atlas 3D (mean 3.25, SD 0.10), Complete Anatomy 2024 (mean 3.28, SD 0.15), and Esqueleto|Anatomía 3D (mean 3.35, SD 0.19). The average scores for section E are listed in Table 3.

Regarding the “perceived effectiveness” section (ie, section F), the recorded mean (SD) was 3.65 (0.18). The 3 top-rated apps with the highest scores were Organos internos 3D (anatomía) (mean 3.93, SD 0.10), VOKA Anatomy Pro (mean 3.90, SD 0.11), and Sistema óseo en 3D (Anatomía) (mean 3.87, SD 0.15). In contrast, the 3 apps with the lowest scores in this section were Complete Anatomy 2024 (mean 3.37, SD 0.20), Anatomy–Atlas 3D (mean 3.40, SD 0.13), and Visual Anatomy Lite (mean 3.45, SD 0.15). The average scores for section F are listed in Table 4.

**Figure 2:**
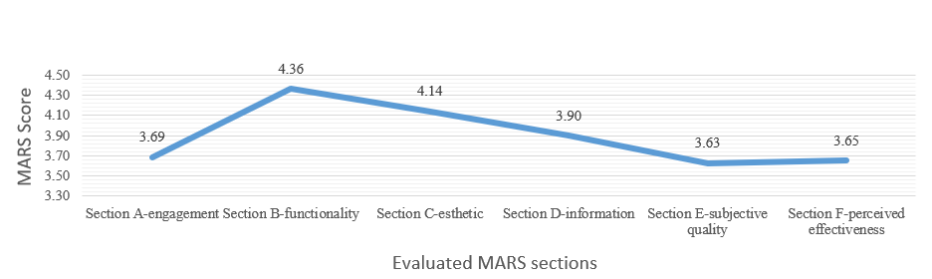
Average scores by the Mobile App Rating Scale (MARS) sections.

The section with the highest score was “functionality” (ie, section B), with a mean (SD) of 4.36 (0.22), followed by “aesthetics” (ie, section C), which scored a mean (SD) of 4.14 (0.21). In the third place was “information quality” (ie, section D), with a mean (SD) of 3.90 (0.22), followed by “engagement” (ie, section A), with a mean (SD) of 3.69 (0.20). The fifth position corresponded to “perceived effectiveness” (ie, section F), with a mean (SD) of 3.65 (0.18), while the sixth and final position was occupied by “subjective quality” (ie, section E), with a mean (SD) of 3.63 (0.22). It is notable that the app-specific score (ie, section F) was higher than the subjective quality score (ie, section E), although the latter was lower than the overall MARS quality score (mean 4.02, SD 0.20). This is demonstrated in Figure 2.

**Table 3:** Average score for “subjective quality” (section E).

App name	Section E—subjective quality (mean 3.63, SD 0.22), mean (SD)
VOKA 3d Anatomy and Physiology	3.95 (0.10)
Organos internos 3D (anatomía)	3.93 (0.17)
Sistema óseo en 3D (Anatomía)	3.88 (0.22)
Anatomy Learning-Anatomía 3D	3.85 (0.10)
Sistema muscular 3D (Anatomía)	3.83 (0.17)
Flashcards de Daily Anatomy	3.78 (0.10)
Teach Me Anatomy	3.75 (0.10)
Anatomyka-Anatomía 3D	3.73 (0.15)
Anatomy by Muscle & Motion	3.63 (0.15)
El cuerpo humano en 3D	3.60 (0.14)
3D Bones and organs (Anatomy)	3.58 (0.17)
Biodigital Human-3D Anatomy	3.55 (0.19)
Gray’s Anatomy-Anatomy Atlas	3.53 (0.13)
e-Anatomy	3.48 (0.17)
Visual Anatomy Lite	3.40 (0.14)
Esqueleto|Anatomía 3D	3.35 (0.19)
Complete Anatomy 2024	3.28 (0.15)
Anatomía–Atlas 3D	3.25 (0.10)

**Table 4:** Average score for “perceived effectiveness” (section F).

App name	Section F—perceived effectiveness (mean 3.65, SD 0.18), mean (SD)
Organos internos 3D (anatomía)	3.93 (0.10)
VOKA 3d Anatomy and Physiology	3.90 (0.11)
Sistema óseo en 3D (Anatomía)	3.87 (0.15)
Anatomy Learning-Anatomía 3D	3.82 (0.21)
Flashcards de Daily Anatomy	3.80 (0.14)
Sistema muscular 3D (Anatomía)	3.78 (0.12)
3D Bones and organs (Anatomy)	3.73 (0.21)
Anatomyka-Anatomía 3D	3.72 (0.08)
Teach Me Anatomy	3.70 (0.17)
Anatomy by Muscle & Motion	3.60 (0.11)
El cuerpo humano en 3D	3.58 (0.08)
Biodigital Human-3D Anatomy	3.57 (0.15)
Gray’s Anatomy-Anatomy Atlas	3.55 (0.22)
Esqueleto|Anatomía 3D	3.48 (0.35)
e-Anatomy	3.47 (0.15)
Visual Anatomy Lite	3.45 (0.15)
Anatomía–Atlas 3D	3.40 (0.13)
Complete Anatomy 2024	3.37 (0.20)

#### Average Scores by Section and Item

The following section details the items that received the highest and lowest scores in the various evaluated sections. In section A, item 5 concerning the target population received a mean score of 3.87 (SD 0.24), whereas item 4 related to interactivity scored a mean value of 3.52 (SD 0.20). In section B, item 6 on performance received the highest score with a mean value of 4.57 (0.14), followed by item 8 related to navigation with a mean score of 4.24 (SD 0.23). In section C, item 11 concerning graphics received a mean score of 4.37 (SD 0.28), and item 12 on visual appeal scored a mean value of 4.02 (SD 0.26). In section D, item 17 on the quality of visual information received a mean score of 3.98 (SD 0.24), whereas item 18 on credibility scored a mean value of 3.83 (SD 0.31). In section E, item 23 on overall quality received a mean score of 3.79 (SD 0.20), and item 22 on willingness to pay for the app scored a mean value of 3.52 (SD 0.24). Finally, in section F, the item with the highest overall mean score was “attitudes,” with a mean value of 3.77 (SD 0.23), whereas the item “help seeking” received the lowest score (mean 3.54, SD 0.23). The mean scores with respect to the section and items are listed in Table 5.

**Table 5:** Mean scores by section and item.

Section and item			Scores, mean (SD)
**Section A—engagement (mean 3.69, SD 0.20)**			
	Item 1. Entertainment	3.67 (0.35)	
	Item 2. Interests	3.78 (0.28)	
	Item 3. Customization	3.59 (0.20)	
	Item 4. Interactivity	3.52 (0.20)	
	Item 5. Target population	3.87 (0.24)	
**Section B—functionality (mean 4.36, SD 0.22)**			
	Item 6. Performance	4.57 (0.14)	
	Item 7. Ease of use	4.37 (0.37)	
	Item 8. Navigation	4.24 (0.23)	
	Item 9. Gestural design of the app	4.27 (0.23)	
**Section C—aesthetics (mean 4.14, SD 0.21)**			
	Item 10. Design	4.04 (0.19)	
	Item 11. Graphics	4,37 (0.28)	
	Item 12. Visual appeal	4.02 (0.26)	
**Section D—quality of information (mean 3.90, SD 0.23)**			
	Item 13. Accuracy of information description	3.86 (0.23)	
	Item 14. Objectives	3.95 (0.25)	
	Item 15. Quality of information	3.87 (0.24)	
	Item 16. Amount of information	3.91 (0.26)	
	Item 17. Quality of visual information	3.98 (0.24)	
	Item 18. Credibility	3.83 (0.31)	
	Item 19. Evidence base	—^a^	
**Section E—subjective quality (mean 3.63, SD 0.22)**			
	Item 20. Would you recommend this app?	3.58 (0.26)	
	Item 21. How many times would you use this app?	3.61 (0.23)	
	Item 22. Would you pay for this app?	3.52 (0.24)	
	Item 23. General qualifications	3.79 (0.20)	
**Section F—perceived effectiveness (mean 3.65, SD 0.18)**			
	Awareness	3.67 (0.27)	
	Knowledge	3.64 (0.22)	
	Attitudes	3.77 (0.23)	
	Intention to change	3.57 (0.22)	
	Help seeking	3.54 (0.23)	
	Behavior change	3.71 (0.17)	

^a^No apps presented explicit scientific support in the descriptions and comments.

#### MARS Overall Quality Scores and Star Rating (Item 23)

The overall MARS quality scores were higher than the scores for item 23 (ie, subjective quality). Similarly, the overall MARS star ratings (ie, item 23) were lower than the star ratings in the Google Play store (Table 6).

**Table 6:** Overall MARSa quality scores, overall star ratings for item 23, and star ratings in the Google Play store.

App name	Health professionals		Users	
	MARS overall quality score (mean 4.02)	MARS (item 23; mean 3.79)	Star rating in the Google Play store (mean 4.63)	Downloads
Organos internos 3D (anatomía)	4.34	4.10	4.90	>5,000,000
Sistema óseo en 3D (Anatomía)	4.32	4.10	4.90	>1,000,000
VOKA 3d Anatomy and physiology	4.29	4.10	4.80	>100,000
Anatomy Learning-Anatomía 3D	4.22	4.00	4.80	>10,000,000
Flashcards de Daily Anatomy	4.18	3.90	4.80	>500,000
Teach Me Anatomy	4.15	3.90	4.70	>1,000,000
Anatomyka-Anatomía 3D	4.12	3.90	4.70	>500,000
3D Bones and organs (Anatomy)	4.04	3.80	4.70	>1,000,000
El cuerpo humano en 3D	4.03	3.80	4.60	>1,000,000
Sistema muscular 3D (Anatomía)	4.03	3.80	4.80	>1,000,000
Biodigital Human-3D Anatomy	3.97	3.70	4.60	>500,000
Anatomy by Muscle & Motion	3.94	3.70	4.60	>500,000
e-Anatomy	3.92	3.70	4.50	>1,000,000
Gray’s Anatomy-Anatomy Atlas	3.85	3.70	4.60	>1,000,000
Esqueleto|Anatomía 3D	3.83	3.60	4.40	>1,000,000
Visual Anatomy Lite	3.80	3.60	4.40	>1,000,000
Complete Anatomy 2024	3.73	3.50	4.30	>1,000,000
Anatomía–Atlas 3D	3.66	3.40	4.30	>1,000,000

^a^MARS: Mobile App Rating Scale.

### Statistical Analysis

#### ICC (Assessment Reliability)

The average reliability measures of the evaluation ranged from “good” to “excellent.” In the engagement section (ie, section A), an ICC of 0.892 (95% CI 0.807-0.952) was obtained. In the functionality section (ie, section B), the ICC was 0.901 (95% CI 0.822-0.956). In the aesthetics section (ie, section C), an ICC of 0.866 (95% CI 0.758-0.941) was recorded. In the information quality section (ie, section D), the ICC was 0.890 (95% CI 0.804-0.951). In the subjective quality section (ie, section E), an ICC of 0.862 (95% CI 0.751-0.939) was obtained. Finally, in the app specificity section (ie, section F), an ICC of 0.868 (95% CI 0.764-0.941) was recorded. Similarly, the reliability of the overall MARS quality evaluation (ie, average of sections A, B, C, and D) was classified as “excellent,” with an ICC of 0.912 (95% CI 0.820-0.963).

#### Pearson Correlation

For the calculation of Pearson correlation coefficient, the average MARS quality scores and the scores for subjective item 23 from section E presented earlier in Table 6 were considered. The result showed an excellent correlation (*r*=0.989, *P*<.001; Table 7). Also, the 95% CI for this correlation was 0.971 to 0.996, based on the Fisher r-to-z transformation.

**Table 7:** Pearson correlation results.

Correlations			MARS overall quality score		MARS (item 23)
**MARS overall quality score**					
	Pearson correlation	1		0.989^a^	
	*P* value (bilateral)	—^b^		<.001	
	N	19		19	
**MARS (item 23)**					
	Pearson correlation	0.989^a^		1	
	*P* value (bilateral)	<.001		—	
	N	19		19	

^a^The correlation is significant at the .01 level (2 sided).

^b^Not applicable.

## Discussion

### Overview

The primary objective of this study was to identify and assess the quality of mobile apps related to human anatomy available on Google Play using the MARS. This scale focuses on the usability and accessibility of mobile health apps, considering aspects such as engagement, functionality, aesthetics, information quality, subjective quality, and app specificity. The MARS organizes the evaluations of the apps into 3 different dimensions. The first dimension includes sections A, B, C, and D and focuses on the evaluation of the objective technical items. The evaluations in the second and third dimensions are subjective and are divided into 2 sections: section E, which considers the evaluator’s personal appreciation, and section F, which focuses on the perceived effectiveness. These 3 dimensions are crucial because, while mobile health apps must meet functionality and design standards, the evaluator’s perception and the app’s impact are determinants for its adoption. In addition, the MARS sections cannot be considered in isolation, as they are interrelated and influence each other.

### Principal Findings

In the first dimension of the MARS, the best-rated section was “functionality” with a mean score of 4.36, followed by “aesthetics” with a mean score of 4.14, “information quality” with a mean score of 3.90, and, finally, “engagement” with a mean score of 3.69, which was the least valued. Although the apps generally received a good average score, it is crucial to examine the relatively low ratings in fundamental aspects such as engagement (mean score 3.69) and look for solutions. In order to strengthen the engagement section (ie, section A), which is made up of items 1 to 5 of the MARS (ie, entertainment, interest, personalization, interactivity, and target population), specific recommendations can be applied for each item. In item 1 (ie, entertainment), it is suggested to integrate elements such as gamification, challenges, achievements, rewards, or progressive levels and use good quality graphics, attractive colors, animations, and multimedia content (eg, videos and music) to make the user experience more attractive. In item 2 (ie, interest), it is recommended to include new content and personalized reminders. For item 3 (ie, personalization), it is recommended that users be able to adjust themes, difficulty levels, colors, or display modes according to their preferences, as well as the use of artificial intelligence to offer suggestions based on the user’s preferences. Regarding item 4 (ie, interactivity), it is suggested to incorporate interactive content such as questionnaires and practical activities that require active participation, as well as real-time communication to forums, live chats, or social interactions to encourage collaboration between users and provide immediate feedback to correct errors or recognize achievements. Finally, in item 5 (ie, target population), it is recommended to carry out previous studies on the characteristics and needs of the target population, such as age, educational level, and cultural context, and ensuring that the content, graphics, and design are consistent with the population for which the app was designed.

The average general quality score according to the MARS (ie, sections A, B, C, and D) was good (mean score 4.02), supported by excellent reliability with an ICC of 0.912 and a 95% CI of 0.820 to 0.963. The mobile apps that excelled in overall quality according to the MARS (ie, sections A, B, C, and D) were Organos internos 3D (anatomía) with a mean score of 4.34, Sistema óseo en 3D (Anatomía) with a mean score of 4.32, and VOKA Anatomy Pro with a mean score of 4.29. These results indicate that these apps, having received high scores and offering high-quality content, can be recommended for users interested in learning human anatomy. In the second and third dimensions of the MARS, corresponding to sections E and F, where the impact on the user is more significant, the lowest average scores were recorded: subjective quality with a mean score of 3.63 and app specificity with a mean score of 3.65. These ratings were even lower than the general quality score of the MARS, which was 4.02.

These results underscore the importance of conducting a thorough analysis of all the 3 dimensions of the MARS; otherwise, apps that are technically well developed might be overvalued, whereas those that receive better subjective ratings from users could be overlooked. This indicates that developers of human anatomy mobile apps should not only address aspects of functionality, aesthetics, engagement, and information but also actively consider user perception and the impact of their apps. There are various practical uses of the study’s results, such as a more appropriate selection of mobile apps in the student context or in medical practice, where those that obtained a higher score in the MARS evaluation are chosen, which provide greater reliability and comfort in use.

Another relevant point of discussion is that the MARS, specifically item 19 (ie, section D), which addresses “information quality,” assesses whether mobile apps have scientific foundations that support their usefulness. However, the apps evaluated in this study did not present explicit scientific support in the descriptions and comments provided by the developers, which is why they lack a rating in item 19 of the MARS evaluation.

Therefore, it is crucial that research centers and universities get involved in the development of mobile health apps so that they are supported by scientific research and can be hosted in app stores to make them accessible to the general public. Collaboration among software developers, health professionals, researchers, and academics in the creation and review of educational materials for a medical mobile app would generate greater confidence in its use. In addition, conducting validation studies in real learning environments also plays an important role in assessing the quality and effectiveness of apps through various methodologies, such as the MARS framework discussed in this study.

### Limitations

The main limitations are the exclusion of paid apps, apps in languages other than English or Spanish, and apps with a star ratings less than 4.3. In addition, the search was limited to apps present in the Google Play store. Although these criteria may seem restrictive, English is the predominant language in global medical education, ensuring that the evaluated apps covered a substantial portion of the app market. However, the exclusions may limit the scope, particularly by omitting paid apps, which in certain cases may offer higher-quality content that could facilitate and enhance the learning of anatomy. To address these limitations in future research, inclusion criteria could be expanded to incorporate human anatomy mobile apps available in other languages or those that are paid, creating a broader repertoire for analysis. This approach would also enable comparative studies, such as exploring potential differences between free apps and those requiring a license or payment.

### Conclusions

This study provides a comprehensive and detailed analysis of apps available for teaching human anatomy, aimed at health care professionals, medical students, and interested users. For example, students and health professionals can both use a human anatomy mobile app before orthopedic surgery to consult a 3D model of the leg of a patient with a femur fracture. This would allow them to more accurately understand the location of bones, blood vessels, and muscles in the affected region, contributing to greater success in the procedure.

Overall, the evaluated apps demonstrated high quality, particularly excelling in functionality and aesthetic design. However, some apps need to improve aspects such as user engagement (ie, section A) and the quality of the information provided (ie, section D). Among the highest-rated apps according to the MARS are Organos internos 3D (anatomía), Sistema óseo en 3D (Anatomía), and VOKA Anatomy Pro.

The subjective MARS score (ie, item 23) was 3.79, in contrast to the average rating of 4.63 given by users on the Google Play store. This suggests that evaluators provided lower ratings, whereas users tend to overrate the apps. This discrepancy may stem from the fact that evaluators typically adhere to more rigorous and objective criteria, systematically assessing technical, functional, and usability aspects. Professional evaluators are often more critical regarding technical implementation and practical utility.

In contrast, users base their ratings on personal and subjective experiences, scoring according to their expectations and the level of satisfaction experienced while using the app. Both perspectives offer valuable feedback: on the one hand, an objective evaluation of quality, and on the other hand, a subjective evaluation of user satisfaction. This difference in ratings does not negatively impact the overall MARS evaluation of the apps. Instead, it provides a perspective where both developers and potential users can identify strengths and areas for improvement from complementary approaches.

This study highlights the evolving role of mobile apps as transformative tools in medical education by offering innovative solutions for accessibility and interactivity in learning. Mobile apps use advanced features such as 3D models, simulations, and dynamic interfaces, and these tools overcome the limitations of traditional methods of teaching human anatomy, such as the scarcity of cadavers and high costs of dissection laboratories. In addition, they facilitate personalized learning of topics and selection of difficulty levels. They allow continuous access, allowing students to practice and reinforce their knowledge anytime, anywhere.

To maximize the impact of mobile apps in medical education, we suggest strategies focused on design and functionality, such as the incorporation of gamification elements, challenges, and rewards to increase user motivation, as well as strengthening interactivity through real-time feedback, collaborative learning tools, and interactive clinical cases. It is essential to align the content of the apps with medical education curricula to ensure their relevance and applicability. Similarly, we recommend combining their use with traditional methods, such as face-to-face classes and laboratory practice, to offer a comprehensive learning experience. Training teachers to integrate these tools into their teaching methodologies is also essential. Finally, to guarantee both the scientific rigor and the accessibility of these mobile apps, we propose collaboration with universities and research centers to develop content based on solid scientific evidence.

## Abbreviations

AR: augmented reality
ICC: intraclass correlation coefficient
MARS: Mobile App Rating Scale
PRISMA: Preferred Reporting Items for Systematic Reviews and Meta-Analyses
STROBE: Strengthening the Reporting of Observational Studies in Epidemiology

## Data Availability

The data used in this study are available upon request from the corresponding author.
